# Rational design of a PKA-based sensor for cGMP

**DOI:** 10.1186/2050-6511-16-S1-A64

**Published:** 2015-09-02

**Authors:** Robin Lorenz, Daniel He, Choel Kim, Chinten J Lim, Friedrich W Herberg

**Affiliations:** 1 Department of Biochemistry, University of Kassel, Kassel, Germany; 2 Department of Pediatrics, University of British Columbia, Vancouver, British Columbia, Canada; 3 Child and Family Research Institute, BC Children's Hospital, Vancouver, British Columbia, Canada; 4 Verna and Marrs McLean Department of Biochemistry and Molecular Biology, Baylor College of Medicine, Houston, TX 77030, USA; 5 Department of Pharmacology, Baylor College of Medicine, Houston, TX 77030, USA

## Background

The cAMP-dependent protein kinase (PKA) and the cGMP-dependent protein kinase (PKG) are highly homologous enzymes differing in their specificity for cAMP and cGMP, respectively. Recent structure-function studies led to the identification of key residues responsible for cGMP-specificity in PKG [1]. Introduction of these amino acids into PKA switches its cyclic nucleotide selectivity [2]. Here we demonstrate that the same mutations turn a cAMP-specific FRET-based A-kinase activity reporter (AKAR) into a cGMP-specific reporter.

## Methods and results

cGMP-specific threonine and arginine residues found in PKG I were introduced into both the N-terminal and C-terminal cyclic nucleotide binding domains (CNB-A and CNB-B) of the PKA regulatory subunit Iα (RIα) using site-directed mutagenesis. The four resulting constructs were: wild type, CNB-A mutant (T192R/A212T), CNB-B mutant (G316R/A336T) and CNB-A/B mutant which has all aforementioned mutations.

While wild type RIα showed a strong selectivity for cAMP vs. cGMP in both in vitro binding and kinase activation studies, either CNB-A or -B mutants bound both nucleotides with equal affinity, resulting in PKA holoenzymes that were activated by both nucleotides equally. The CNB-A/B mutant had a significantly higher affinity for cGMP, and the corresponding PKA holoenzyme was activated selectively by cGMP.

Mouse embryonic fibroblasts derived from a PKA RIα knockout mouse were contransfected with the RIα constructs along with an AKAR to test their responsiveness for the cell-permeant analogs 8-CPT-cAMP and 8-CPT-cGMP, respectively [3]. Our results showed that both the wild type and the CNB-B mutant do not respond to 8-CPT-cGMP. In contrast, the CNB-A mutant showed a phasic response to 8-CPT-cGMP, and the response of the double mutant CNB-A/B sustained longer.

## Conclusion

Cyclic nucleotide-dependent regulation of protein kinase activity is an important aspect of eukaryotic signal transduction. The vice versa specificity of PKA and PKG determines the fidelity of cAMP-PKA and cGMP-PKG pathways. We applied our understanding of cyclic nucleotide selectivity to cellular sensor design and showed that mutating four key residues within a cAMP-specific reporter switches it into a cGMP-specific reporter.

Cyclic nucleotide selectivity has apparently evolved through mutations in the CNB. The identification of these key residues will improve the design of cyclic nucleotide-selective cellular reporters.
